# Early growth response-1: Key mediators of cell death and novel targets for cardiovascular disease therapy

**DOI:** 10.3389/fcvm.2023.1162662

**Published:** 2023-03-28

**Authors:** Yixin Xie, Yongnan Li, Jianshu Chen, Hong Ding, Xiaowei Zhang

**Affiliations:** ^1^ Department of Cardiology, Lanzhou University Second Hospital, Lanzhou, China; ^2^ Department of Cardiac Surgery, Lanzhou University Second Hospital, Lanzhou, China

**Keywords:** early growth response-1, cell death, apoptosis, autophagy, cardiovascular diseases, transcription factors

## Abstract

**Significance:**

Cardiovascular diseases are seen to be a primary cause of death, and their prevalence has significantly increased across the globe in the past few years. Several studies have shown that cell death is closely linked to the pathogenesis of cardiovascular diseases. Furthermore, many molecular and cellular mechanisms are involved in the pathogenesis of the cardiac cell death mechanism. One of the factors that played a vital role in the pathogenesis of cardiac cell death mechanisms included the early growth response-1 (*Egr-1*) factor.

**Recent Advances:**

Studies have shown that abnormal *Egr-1* expression is linked to different animal and human disorders like heart failure and myocardial infarction. The biosynthesis of *Egr-1* regulates its activity. *Egr-1* can be triggered by many factors such as serum, cytokines, hormones, growth factors, endotoxins, mechanical injury, hypoxia, and shear stress. It also displays a pro-apoptotic effect on cardiac cells, under varying stress conditions. EGR1 mediates a broad range of biological responses to oxidative stress and cell death by combining the acute changes occurring in the cellular environment with sustained changes in gene expression.

**Future Directions:**

The primary regulatory role played by the *Egr-1*-targeting DNAzymes, microRNAs, and oligonucleotide decoy strategies in cardiovascular diseases were identified to provide a reference to identify novel therapeutic targets for cardiovascular diseases.

## 1. Introduction

**Figure 1:**
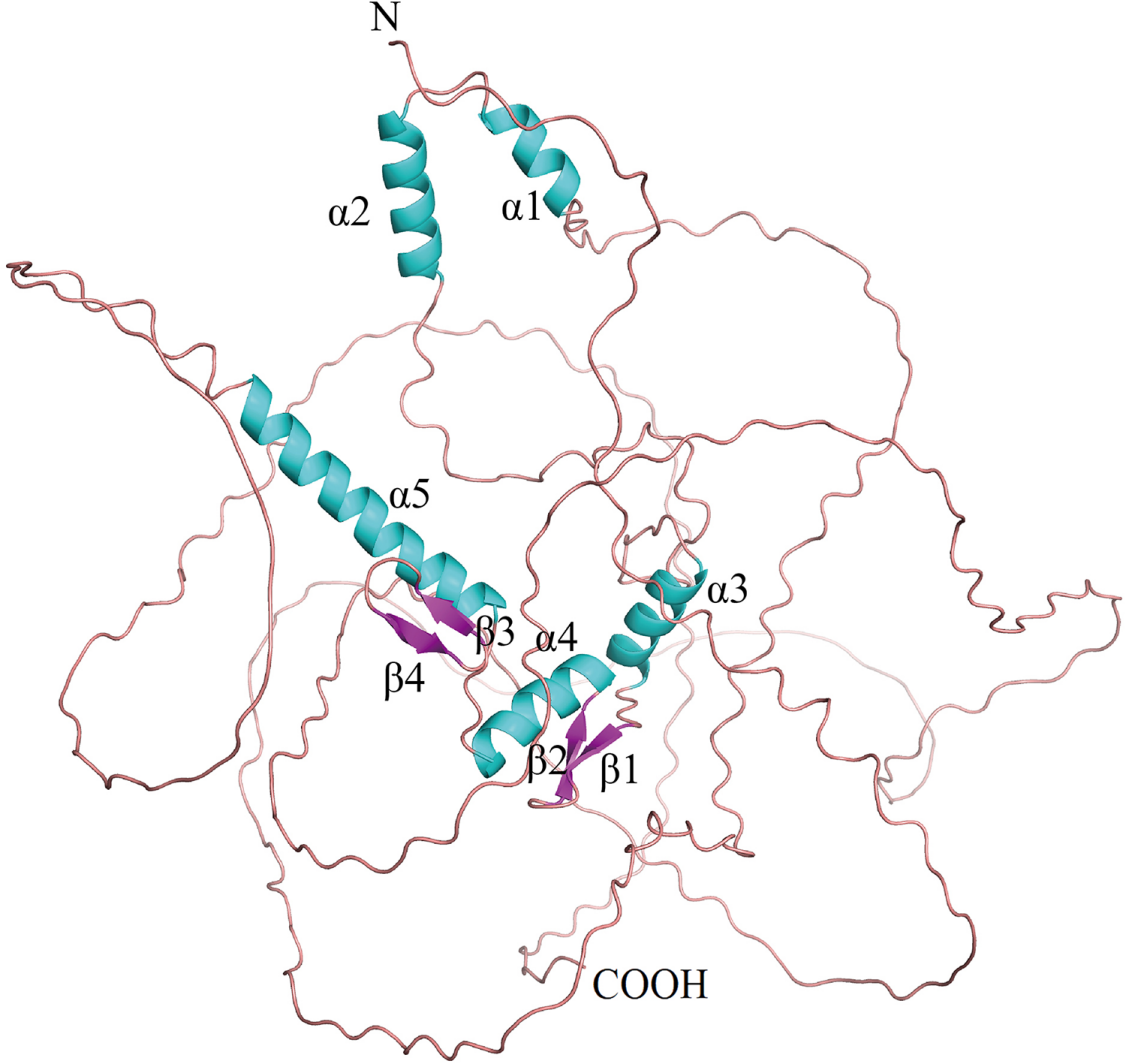
Structure diagram of Egr-1.

Early growth response factor 1 (*Egr-1*) is an early gene, belonging to the EGR family that codes for a Cys_2_-His_2_ zinc finger protein (1). It is located on human chromosome 5q23-q31 (2). EGR1, also called NGFI-A (3), AT225, ZIF268 (4), TIS8, G0S30, KROX-24, and ZNF225, is an 80 kDa DNA-binding transcription factor with 543 residues that regulates transcription. EGR1 contains a three Cys_2_-His_2_ subtype zinc finger structure, an activation regulatory region, as well as a suppression regulatory region, which are located between 332 and 416 amino acids, close to the carboxyl terminus (5). It specifically identifies and binds to the target genes, and regulates their transcription. The *Egr-1* promoter includes serum response elements, which preferentially bind to the GC-rich elements. They also regulate the interaction between different growth factors and this sequence to initiate the *Egr-1* gene expression *via* different mechanisms that involved a co-activator and co-repressor (6). EGR1 binds to the DNA motifs [with a sequence of 5′-GCG(T/G) GGGCG-3′] with the help of the Cys_2_-His_2_-type zinc fingers. The C-terminal zinc finger binds to the 5′-GCG motif, while the majority of N-terminal zinc fingers bind to the 3′-GCG motif, and the middle zinc finger interacts with the middle TGG motif (Figure 1). *Egr-1* gets activated (rapidly and transiently) in different human cell types in response to varying agonist and environmental factors (7). *Egr-1* is triggered by a variety of stimuli, such as serum, cytokines, hormones, growth factors, endotoxins, mechanical damage, hypoxia, and shear stress (8–11). The products of the *Egr-1*-activated target genes play key roles in cell proliferation, differentiation, mitosis, and cell death pathways (12). *Egr-1* expression is associated with many factors linked to cardiovascular pathologies such as atherosclerosis, cardiac hypertrophy, intimal thickening after acute arterial injury, and angiogenesis (13–15). *Egr-1* also plays a role in doxorubicin-induced cardiomyopathy. Some rat model-based studies have shown that *Egr-1* gene inhibition decreases the pathological effects of acute myocardial infarction (AMI) (16, 17). The *Egr-1* phenotypes are seen to be cell type-specific, and *Egr-1* overexpression stimulates cell apoptosis. Additionally, researchers have used catalytic and non-catalytic nucleic acid methods such as DNAzymes, microRNAs, and oligonucleotide decoys in animal models in conjunction with *Egr-1*-deficient mice to obtain novel insights into the regulatory role played by *Egr-1* in cardiovascular diseases. Thus, *Egr-1* can serve as a unique target for therapeutic intervention.

## 2. *Egr-1* is involved in cardiac cell death

### 2.1. *Egr-1* and apoptosis

*Egr-1* regulates the expression of many genes in cardiac cells (18). Studies have shown that *Egr-1* causes cardiac cell apoptosis through various mechanisms. In comparison to necrosis, apoptotic cells are immediately identified and cleared by adjoining phagocytes to prevent inflammation. Apoptosis affects the cardiovascular system and leads to the onset and progression of many cardiovascular disorders. It also helps in clearing the non-myocyte components and cardiomyocytes, which cause heart failure (19) (Table 1).

**Table 1:** Egr-1 is involved in cardiac cell death.

Cell death mechanisms	Cell/animal models	Related pathways/genes	Pathological relevance	Representative studies
Apoptosis	N/A	Bim, Bax, Ppp1r13b, Siva-1	Cardiomyopathy	Cook et al. (20)
				Zhao et al. (21)
				Zins et al. (22)
	LDLR^–/–^ mini-pig model of advanced atherosclerosis, vascular injury (carotid ligation) and inducible plaque rupture (ligation and cuff) in mice.	ERK-ELK1- EGR1 pathway	Advanced atherosclerotic lesion	Fasolo et al. (23)
	CRISPR/Cas9 strategy to introduce a homozygous Ser26 > Ala mutation into endogenous Egr­1 in human vascular endothelial cells	ERK-1 phosphorylate the serine residue at the 26th position in Egr-1	angiogenesis	Santiago et al. (24)
	Male C57BL/6 mice: I/R injury models	EGR1/TLR4/TRIF pathway	AMI	Huang et al. (25)
Autophagy	Male SD rats constructed a CME model by injecting plastic microspheres into the left ventricle	EGR1/BIM/Beclin-1 pathway	Coronary microembolization-induced myocardial injury	Wang et al. (26)
	Cardiomyocytes from newborn SD rats in an I/H environment	EGR1/ BIM /Beclin-1 pathway	Microvascular obstruction	Su et al. (27, 28)
	The left anterior descending artery was ligated followed by reperfusion. cultured cardiomyocytes following H/R injury	EGR1/mTORC1/TFEB pathway	AMI	Huang et al. (29)
	C57BL/6 J mice: I/R injury models, *in vitro* H/R models	MEK/ERK/ EGR1 pathway	AMI	Wang et al. (30)
MPOS	N/A	Overexpression of ANT1 activates Egr-1 expression and induces aggresomes	Mitochondrial myopathy and cardiomyopathy	Liu et al. (31)
Metabolic homeostasis	MPC1^fl/fl^ and EGR1^−/−^ mice, ventricular cardiomyocytes from newborn rat hearts were transduced with Ad-Cre-GFP virus	Egr-1 negatively regulates NCX1 and inhibits the expression of CSQ	Cardiac arrest and death	Wang et al. (32)
				Kasneci et al. (33)
				Nemani et al. (34)
	Male SD rats were used to establish diabetic model	CCN1/ERK1/2/EGR1 pathway	Diabetic cardiomyopathy	Wang et al. (35)
Oxidative stress	oxygen-glucose deprivation/reoxygenation model of myocardial I/R injury using H9c2	Silencing of Egr-1 suppressed the expression of TF and ICAM-1	AMI	Zhao et al. (36)
	Hearts were isolated from fetal rats/ H9c2, reatments with norepinephrine and ROS inhibitors	ROS resulted in an increase in PKC*ε* promoter methylation at Egr-1 and Sp-1 binding sites	AMI	Xiong et al. (37)
	Human aortic smooth muscle cells, treatment with hemin	Egr-1 was mediated by the ROS/ERK/Elk-1 pathway and NF-*κ*B	Inflammatory vascular diseases such as atherosclerosis.	Hasan et al. (38)
Ferroptosis	C57BL/6 mice were used to establish AMI models	EGR1/miR-15a-5p/GPX4 axis	AMI	Fan et al. (39)

MPOS, mitochondrial precursor overaccumulation stress; I/R, Ischemia/reperfusion; H/R, hypoxia/reoxygenation; I/H, ischemia/hypoxia; SD, Sprague-Dawley; AMI, Acute myocardial infarction; ROS, Reactive oxygen species; ANT1, Isoform 1 of adenine nucleotide translocase; NCX1, sodium-calcium exchanger-1; CSQ, Calsequestrin; TF, tissue factor; MPC1, monocyte chemoattractant protein 1; ICAM-1, intercellular cell adhesion molecules 1; GPX4, Glutathione Peroxidase 4.

EGR1 can promote apoptosis by binding and stimulating the levels of different promoters of the apoptosis-based factors, like BIM (Bcl-2 Like 11), BAX (BCL2 Associated X, Apoptosis Regulator), ASPP (Apoptosis Stimulating P53 Protein), and SIVA1 (SIVA1 Apoptosis Induced Factor). The proteins encoded by *Bax* and *Bim* genes belong to the BCL2 protein family. The BCL2 family of proteins forms heterodimers or homodimers and participates in a variety of cellular activities as anti-apoptotic or pro-apoptotic regulators (40–42). On the other hand, *Ppp1r13b* (protein phosphatase 1 regulatory subunit 13b) encodes for ASPP that regulates cell death (43). *Siva-1* encodes for an E3 ubiquitin ligase enzyme that regulates cell proliferation, cell cycle progression, and apoptosis (44).

Mitochondria are known to be key sites for the integration of pro-apoptotic and anti-apoptotic proteins in cardiac cells (45). Studies have shown that upregulated EGR1 can bind to the *Bax* and *Bim* promoters for increasing the BAX and BIM protein levels in cardiac cells that are stressed by injury (46–48). BAX, BIM, and other pro-apoptotic proteins bind to the mitochondrial membrane and interact with mitochondrial Voltage-dependent anion channels (VDAC) to increase the channel opening rate. This further increases membrane permeability, release of cytochrome (Cyt) into the cytoplasm, activation of the Caspase family and metabolic hydrolases, and finally promotes apoptosis (49). BAX forms heterodimers with the BCL2 protein, which inhibits apoptosis and autophagy and acts as an activator of apoptosis. The binding and ratio of the BAX and BCL2 proteins determine the death or survival of cells after apoptosis stimulation (20). BAX level is regulated by *Tp53*/*p53* (tumor protein p53) and it is seen to participate in *Tp53-*mediated apoptosis (50, 51). EGR1 is involved in the upstream transcriptional regulation of Tp53 (52). It affects TP53 level *via* the *Tp53* promoter, and then TP53 activates EGR1 to form a feedback loop (53, 54). On the other hand, *Egr-1* activates the MAPK (Mitogen-Activated Pprotein Kinase)-ELK1 (ETS Transcription Factor ELK1)-EGR1 pathways to promote P21 (Cyclin-Dependent Kinase Inhibitor 1A) level without TP53. Studies have shown that *Tp53* primarily targets P21, which regulates the cell cycle, promotes DNA repair, and induces apoptosis (55, 56).

*Egr-1* specifically targets ASPP, as it plays a crucial role in the immediate up-regulation of ASPP. Additionally, it maintains the basic expression of the *Ppp1r13b* gene during non-stress conditions (21). Furthermore, *Ppp1r13b* stimulates EGR1 protein levels within a positive feedback loop. Initially, EGR1 is activated by multiple stimuli, and then, it binds to EBS, which is located in the *Ppp1r13b* promoter region, thereby transactivating the expression of *Ppp1r13b* in the nucleus. Elevated ASPP level were primarily localized in the cytoplasm, which inhibited the proteasome-mediated degradation of EGR1 and promoted a nuclear import of EGR1. Activated EGR1 can also promote apoptosis by transactivating the proapoptotic targets, such as *Egr-1* itself. In an earlier study, the researchers identified a novel EGR1/ASPP inter-regulatory loop and determined the proapoptotic function of cytoplasmic ASPP by stabilizing EGR1 and inhibiting the autophagy promoter ATG5 (Autophagy protein 5) -ATG12/ATG16 (57, 58). The *Tp53* controls TP53 by increasing its DNA binding and transactivation capabilities on proapoptotic gene promoters. *Ppp1r13b* interacts with the *Tp53* gene and plays a crucial role in regulating apoptosis.

Studies have shown that during the *in vitro* apoptosis induction phase of cardiac fibroblasts, the continuous *Egr-1* expression causes the downstream transcriptional regulation of the pro-apoptotic gene, i.e., *Siva-1*. Normal cardiac fibroblasts do not express the *Siva-1* (22, 59). The *Siva-1* gene codes for the SIVA1 protein that binds to the cytoplasmic tail of the members of the tumor necrosis factor (TNF) receptor superfamily (such as CD27 and a glucocorticoid-induced TNF receptor) that are activated by ligands (60). This protein also contains a domain that is similar to the death domain. It has been demonstrated that *Siva-1* mediates proliferating cell nuclear antigen (PCNA) ubiquitination in response to ultraviolet-induced DNA damage and triggers CD27-mediated apoptosis. SIVA1 is a transcriptional target of the *Tp53* gene, and it induces the oxidative stress-induced apoptosis process (61). Additionally, the *Siva-1* gene encodes for an E3 ubiquitin ligase that suppresses the anti-apoptotic activity of BCL2, leads to caspase-dependent apoptosis, localizes in the mitochondria, and regulates cell cycle progression, cell proliferation, and apoptosis (62, 63).

*Egr-1* controls the level of hundreds of proteins in cardiac cells and implements the above-mentioned processes to bind to different apoptosis-related factor promoters. One important functional switch is the phosphorylation of EGR1. Santiago et al. found that ERK1 (Mitogen-Activated Protein Kinase 3) could phosphorylate the serine residue at the 26th position (Ser26) in EGR1 in human vascular endothelial cells, which has a protective effect against apoptosis. If Ser26 is mutated, endothelial cells will undergo apoptosis (24). In one study, Fasolo et al. determined the effect of the long non-coding RNA myocardial invasion-associated transcript (MIAT) on advanced atherosclerotic lesions. They discovered that *Egr-1* controlled the proliferation and death of smooth muscle cells (SMCs). They also noted that MIAT controlled the proliferation and apoptosis of the SMCs in the carotid artery *via* the ERK- ELK1-EGR1 pathway (23). Studies have found that during myocardial ischemia/reperfusion (I/R), the up-regulated *Egr-1* gene activates TLR4 (Toll Like Receptor 4)/TRIF (TIR Domain Containing Adaptor Molecule 1) signaling pathway, increases neutrophil recruitment, intensifies cell apoptosis, and further aggravates cardiac function injury (25). Another study showed that *Egr-1* could be inhibited by the JAK (Janus Kinase)/STAT (Signal Transducer And Activator Of Transcription) pathway, which decreased the myocardial I/R injury (64).

### 2.2. *Egr-1* and autophagy

*Egr-1* not only contributes to apoptosis but also autophagy. The regulation of cardiomyocyte homeostasis is enhanced by autophagy, which is crucial for cardiac physiology (65). Myocardial ischemia-related cell damage can be reduced by autophagy. It has been demonstrated that inhibiting autophagy would exacerbate heart hypertrophy in patients. In general, autophagy promotes the survival of cells by accelerating their metabolic cycle and allowing their adaptation to their surroundings. However, autophagy of the majority of organelles and cytoplasm in the phagocytes results in cell death. In patients with heart failure, excessive autophagy results in type II cell death, or autophagic cardiomyocyte death (66). Cardiomyocyte death following I/R damage is correlated to impaired autophagy flux. In an earlier study, the researchers noted an elevated EGR1 level in a coronary microembolism rat model, which led to the conclusion that *Egr-1* helps in regulating autophagy and apoptosis (26). Cytoplasmic components, such as protein aggregates, damaged organelles, and lipid droplets, were encapsulated by the double-layer membrane vesicles to form autophagosomes, which could fuse with the lysosomes to form autophagic lysosomes, degrade the enclosed contents, and assist their recycling (67).

**Figure 2:**
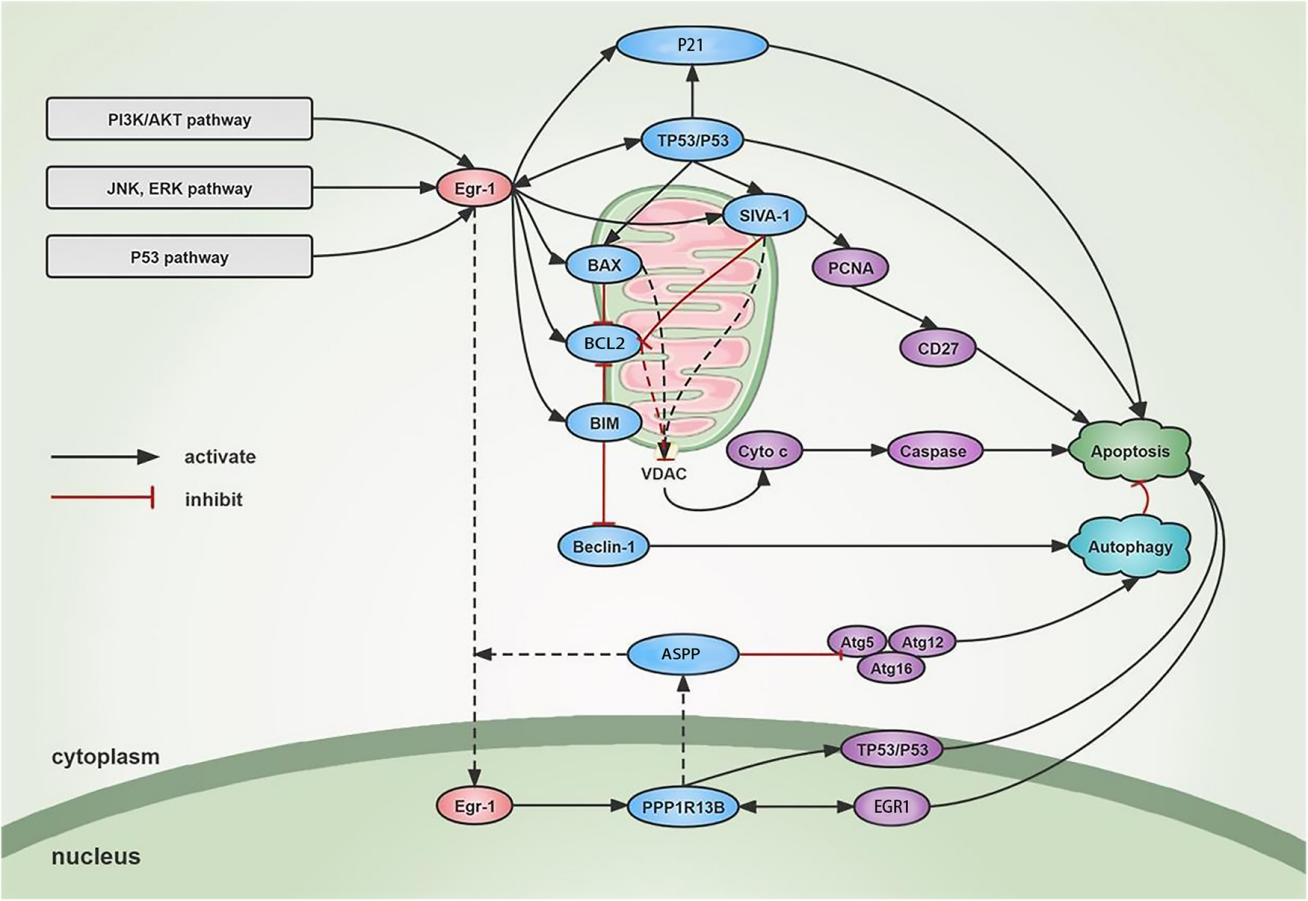
Mechanism of Egr-1 on apoptosis and autophagy. *Egr-1* is activated through different signaling cascades. On one hand, *Egr-1* stimulates the binding of pro-apoptotic proteins such as BIM, BAX, and SIVA1 to the mitochondrial membrane, increases the opening of VDAC, leads to increased membrane permeability, releases cytochrome, and activates Caspase family and metabolic hydrolases to promote apoptosis. SIVA1 can also mediate the ubiquitination of PCNA and induce CD27-mediated apoptosis. *Egr-1* also transactivates the expression of Ppp1r13b in the nucleus. The increased ASPP is mainly localized in the cytoplasm and promotes the nuclear import of EGR1 to promote apoptosis. At the same time, ASPP binds to ATG5-ATG12 and inhibits the binding of ATG5-ATG12 to ATG16 and the formation of ATG16 complex, thus inhibiting cytoprotective autophagy and inducing apoptosis. On the other hand, *Egr-1* can activate TP53 in a feedback loop to further activate EGR1 to induce apoptosis. In addition, *Egr-1* can induce the P21 and promote cell apoptosis independently or dependently on TP53. Autophagy and apoptosis interact with one another, and *Egr-1* can inhibit Beclin-1 through activating BIM and finally inhibit autophagy of cardiac cells.

MicroRNAs (miRNAs) regulate and interact with autophagy and apoptosis. Earlier studies have shown that numerous cardiovascular disorders are related to the regulation of EGR1 by miRNAs (68). It was also concluded that miRNAs control EGR1 for mediating autophagy in cardiac cells. Furthermore, a few independent studies showed that BIM suppresses autophagy independent of its pro-apoptotic activity (69). The findings also showed that BIM performs a dual role in suppressing autophagy and promoting apoptosis. BIM directly interacts with the key autophagy regulator, BCL1, to inhibit autophagy (27, 28); which indicates that it could be involved in the pathogenesis of the disease. When cardiac cells are triggered by different environmental factors, the EGR1/BIM/Beclin-1 pathway is activated, thus inhibiting myocardial autophagy and inducing cell death. Furthermore, when an *in vitro* model of myocardial I/R injury and hypoxia/reoxygenation (H/R) was studied, the researchers noted that circRNAs influenced autophagy by regulating EGR1. Silencing circZNF512 attenuated its ability to bind to miR-181d-5p, and targeted 3′-UTR, thereby impairing EGR1 production, increasing cardiomyocyte autophagy, and inhibiting apoptosis, thus decreasing the myocardial tissue damage. The crosstalk between circZNF512, miR-181d-5p, and EGR1 activated the mTOR (Mechanistic Target Of Rapamycin Kinase)C1/TFEB (Transcription Factor EB) signaling pathway and elevated the mTORC1 level, while the TFEB level was decreased. Furthermore, it was seen that CircZNF512-mediated miR-181d-5p suppression restricted the cardiomyocyte autophagy and increased the myocardial I/R damage (29), with the help of the EGR11/mTORC1/TFEB-based mechanism. Additionally, ERK1/2 refers to a crucial upstream signal molecule that regulates *Egr-1* expression and forms the basis for myocardial I/R. Inhibiting *Egr-1* expression can lessen the MEK/ERK activation-induced myocardial I/R damage (30) (Figure 2).

### 2.3. *Egr-1* and other mechanisms of cell death

According to earlier research, mitochondrial precursor overaccumulation stress (mPOS), which is defined as the dangerous accumulation of unimported mitochondrial proteins in the cytosol, kills cells by a mechanism independent of bioenergetics (70). Liu et al. observed that the overexpression of mitochondrial carrier proteins, notably isoform 1 of adenine nucleotide translocase (ANT1), could lead to the development of numerous cytoplasmic attackers that contained the unimported mitochondrial proteins (31). Dilated cardiomyopathy was linked to the expression of extremely unstable variants of ANT1-induced aggregation and the overexpression of ANT1. It was noted that the *Egr-1* gene was maximally upregulated in response to ANT1 overexpression (71–73). Therefore, different mitochondrial stress factors could activate the nuclear transcription factor, EGR1, *via* ANT1 overexpression, which further increased the mPOS-related cell-killing mechanism, thereby causing cardiomyopathy (74).

In addition to mPOS, metabolic homeostasis (especially calcium homeostasis) can also lead to cardiomyocyte death. Calcium is transferred outside the cardiomyocytes *via* the sodium-calcium exchanger-1 (NCX1), where they bind to calsequestrin (CSQ) and are re-stored in the sarcoendoplasmic reticulum. In the past, researchers have reported that *Egr-1* negatively regulates NCX1 level, which helps in controlling calcium homeostasis, both *in vivo* and *in vitro*. Subsequently, it was discovered that *Egr-1* mostly inhibits CSQ level. Since CSQ is primarily responsible for the storage and release of calcium ions, CSQ inhibition can harm cardiomyocytes and impair cardiac function. The researchers also found that diabetic cardiomyopathy patients experienced issues related to calcium balance and mitochondrial malfunction, which caused the loss of cardiac cells. Inhibiting the production of EGR1 and controlling mitochondrial calcium homeostasis are two mechanisms by which the MOTS-c (Mitochondrial Open Reading Frame Of The 12S rRNA-c) can stop cardiomyocyte death. The above-mentioned calcium homeostasis is not the only factor that influences cardiomyocyte death; mitochondrial metabolic homeostasis is also important. In cardiomyocytes, Ca^2+^ ions enter the mitochondria *via* the mitochondrial Ca^2+^ (MCU), which stimulates the tricarboxylic acid (TCA) cycle to increase ATP production (32, 33, 35, 75–78). An *Egr-1*-mediated gene that codes for MICU1 (Mitochondrial Calcium Uptake 1), the gatekeeper of the mitochondrial calcium uniporter, is transcriptionally upregulated by the mitochondria in response to metabolic balance. After analyzing the MCU-mediated mitochondrial matrix Ca (_m_Ca) uptake during metabolic stress, it was noted that *Egr-1* regulates the *Micu1* promoter, induces MICU1 level during mitochondrial stress, inhibits basal _m_Ca accumulation, and lowers mitochondrial bioenergetics, thereby, preventing _m_Ca overload and ensuing cardiac cell death (34).

Cardiomyocyte death and oxidative stress are also closely related. Oxidative stress negatively affects the development of ischemic heart disease, which leads to irreversible damage and even death of myocardial cells (79). *Egr-1* is a redox-sensitive gene and is involved in the pathophysiology of cardiovascular diseases (80–83). Oxidative stress occurs when cellular reductases cannot protect the cells from increased Reactive oxygen species (ROS), which induce EGR1 protein level. This further imbalanced the redox state, which increased DNA and protein damage. Furthermore, higher concentrations of the oxidants can activate different signaling pathways, which, in turn, target the promoters of “redox-sensitive” genes. Notably, heart failure is characterized by the activation of the sympathetic nervous system, which increases oxidative stress in the cardiovascular system (84, 85). Protein kinase C*ε* (*PKCε*) plays an important role in cardioprotection, where the *PKCε* gene inhibition increases susceptibility to cardiac I/R injury (86). *Egr-1* exhibits a regulatory effect on PKC*ε* (37). In their study, Xiong et al. found that norepinephrine-induced ROS increased the methylation of the Egr-1 and Sp-1 binding sites in the *PKCε* promoter, which led to *PKCε* repression, finally, leading to cardiac function impairment (87). Also, it has been noted that free heme is one of the main causes of ROS in the cardiovascular system. Free heme is released from hemoglobin due to bleeding or hemolysis, leading to oxidative stress and cell death (88, 89). Hasan et al. observed that Hemin (oxidized heme) upregulates the vascular smooth muscle cells (VSMCs) in a redox-sensitive manner *via* the ROS/ERK and NF-*κ*B (Nuclear Factor Kappa B) pathways, which results in thrombotic or atherosclerotic lesions (38). Also, it has been discovered that *Egr-1* inhibition can shield cardiac cells from oxygen-glucose deprivation/reperfusion (OGD/R)-induced injury (36). Studies also showed that dimethyl fumarate protects the cardiac cells against myocardial I/R injury by blocking NOX-4 (NADPH oxidase 4)-mediated ROS production. A few researchers stated that *Egr-1* interacts with two additional mechanical stress-related MAPKs, including JNKs. These JNKs, sometimes called stress-activated protein kinases or P38 subtypes, are all activated due to mechanical, oxidative, or environmental stress (90).

In 2012, the researchers identified ferroptosis as a novel cell death mechanism, which was characterized by excessive intracellular lipid peroxide concentrations (91). GPX4 (Glutathione Peroxidase 4) is considered a major factor that inhibits ferroptosis, as it protects cell integrity by eliminating cellular lipid peroxidation and maintaining the balance of the intracellular redox state. Several recent studies have demonstrated that ferroptosis plays a vital role in the development of cardiovascular diseases, such as myocardial infarction, cardiomyopathy, myocardial I/R injury, and heart failure (92). A few findings revealed that GPX4 level was significantly decreased during the early and middle myocardial infarction stages. The researchers also noted that silencing *GPX4* could induce ferroptosis of myocardial cells (93). Ferroptosis is characterized by intracellular redox imbalance. A few earlier reports indicated that *Egr-1* could be involved in oxidative stress injury, and it was hypothesized that *Egr-1* may be involved in the development of ferroptosis. Studies were conducted using an *in vitro* ferroptosis model, and the results indicated that patients with AMI displayed an increased *Egr-1* expression (39). *Egr-1* inhibition decreased the miR-15a-5p level and elevated the GPX4 level. It further increases SOD (Superoxide Dismutase) activity, lowers ROS level, and decreases MDA (malondialdehyde) levels and cardiac cell death rates. These findings imply that miR-15a-5p expression is regulated by *Egr-1* downregulation, which inhibits ferroptosis.

## 3. *Egr-1* plays a vital role in the pathogenesis of cardiovascular disease

*Egr-1* plays a crucial role in cardiovascular biology, and its expression is related to several aspects of cardiovascular pathology. *Egr-1* can promote or reduce the synthesis of many pro-inflammatory and anti-inflammatory protein mediators that bind to the complimentary motifs on the DNA of the gene of interest, and are involved in the cell death mechanisms. These mediators primarily regulate angiogenesis, which helps in the healing and regeneration of injured tissues under physiological conditions. It was reported that these mediators actively promote tissue destruction under pathological conditions. According to earlier findings, the *Egr-1* was involved in the pathogenesis of atherosclerosis, from the development of foam cells to the onset of acute cardiovascular and cerebrovascular ischemia events. *Egr-1* is also believed to be a possible aggregator of other heterogeneous atherosclerotic risk factors, including hyperlipidemia, aberrant hemorheology (observed in hypertension), and other infectious factors. *Egr-1* is overexpressed during AMI, which reduces cardiomyocyte energy loss and mass cardiomyocyte death. Targeting *Egr-1* can decrease the degenerative effects of AMI in rats. *Egr-1* also mediates doxorubicin-induced cardiomyopathy. Therefore, it is important to understand the *Egr-1* regulation mechanism, as it could help in the future treatment of cardiovascular diseases.

### 3.1. MiRNAs regulation of *Egr-1* mRNA

MiRNAs are small non-coding RNAs that are involved in gene regulation. Recent studies have revealed that many miRNAs regulate vascular homeostasis and play unique regulatory roles in cardiovascular diseases (94–96). The analysis of dysregulated gene expression in miR-208a mutant mice revealed higher EGR1 and FOS (Fos Proto-Oncogene) levels in the heart. This finding suggests that miR-208 regulates the *Egr-1* gene response to cardiac stress. MiR-499 was significantly enriched in the human heart ventricles during microRNA screening (97). Also, substantial changes were noted in miR-499 levels in heart samples collected from aortic stenosis patients with heart failure and pressure overload (98). Furthermore, miR-499 transgenic mice experienced or were susceptible to cardiac dysfunction. *Egr-1* is a critical component of the transcriptional response hierarchy to cardiac stress (99), which may explain why miR-499 levels significantly affect the *Egr-1* gene response. Recent research has demonstrated that the *Egr-1*-mediated miR-99 could be regarded as a critical factor in AKT1 (AKT Serine/Threonine Kinase 1) level, which, in turn, controls important cell death pathways involved in the transformation of normal hypertrophy into pathological hypertrophy (100). EGR1 is a key transcriptional activator in the pathological hypertrophy process that might promote *PTEN* (Phosphatase And Tensin Homolog), which is a negative regulator of AKT (101, 102). EGR1 regulates AKT *via* two mechanisms: post-transcriptionally *via* the miR-99 family and post-translationally *via PTEN*. *Egr-1* knockdown converts pathological hypertrophy to physiological hypertrophy by activating the AKT pathway. The miR-99 family regulates Akt/mTOR/IGF1 (Insulin Like Growth Factor 1) *via Egr-1*-mediated expression, which governs both pathological and healthy hypertrophy (103). *AKT1* silencing reduces EGR1 phosphorylation, which decreases miR-99 transcription, resulting in lower apoptosis and cytotoxicity levels. Plasma cholesterol influences a variety of cardiovascular diseases, including atherosclerosis and coronary artery disease (104). Since miR-27a regulates the cholesterol production pathway, it could be hypothesized that a putative EGR1 binding site existed near the miR-27a promoter region. Computational and experimental studies have demonstrated that *Egr-1* regulates miR-27a at basal and high cholesterol levels (105). Overexpression of miR-15a-5p promotes ferroptosis in cardiomyocytes, which, in turn, exacerbates hypoxic injury. In the case of AMI, suppressing *Egr-1* can limit miR-15a-5p levels, boost GPX4 protein level, and decrease ferroptosis and myocardial damage (39). Recent evidence suggests that miR-146a inhibits *Egr-1* transcription and expression, *in vivo* and *in vitro*, and attenuates AMI-induced myocardial injury through the TLR4/NF-*κ*B pathway (106).

### 3.2. DNAzymes target *Egr-1* mRNA in cardiovascular diseases

DNAzymes are a new generation of catalytic oligodeoxynucleotides that can be used as an effective gene-silencing strategy. They can be used for overcoming the disadvantages of oligonucleotides and ribozymes and present an effective *in vivo* gene targeting technique (107). These agents break phosphodiester linkages between specific purines and pyrimidines by precisely base-pairing and de-esterifying the DNAzymes with the target mRNA. Studies have shown that DNAzymes that target *Egr-1* are biologically effective in treating AMI and other cardiovascular conditions (108). The proliferation of Endothelial cells (ECs) and VSMCs, in addition to the neointimal formation, is inhibited by *Egr-1*-targeting DNAzymes (109). *Egr-1* also plays a significant role in the injury responses displayed by other cells related to the cardiovascular system (110). ED5 was the first DNAzyme that was successfully tested using an animal model (111). It was noted that ED5 inhibited the EGR1 level and regulated the cardiovascular diseases caused by intimal thickening after permanent carotid artery ligation in rats. The data indicated that ED5-mediated *Egr-1* downregulates EGR1 after myocardial I/R and attenuates the level of the myocardial intercellular cell adhesion molecules (ICAM) -1 and neutrophil adhesion during myocardial injury and oxidative stress in cardiomyocytes. ED5 also mediates *Egr-1*-related inhibition of cyclin D1, monocyte chemoattractant protein (MCP) -1, tissue factor (TF), cyclin-dependent kinase 4 (CDK4), macrophage inflammatory protein (MIP) -2, and Plasminogen activator inhibitor (PAI)-1 (112). Inhibition of these downstream targets can decrease the cell death rate caused by myocardial ischemia (11). Thus, intracoronary inhibition of *Egr-*1 by targeted DNAzymes may reduce the size of the infarct by modulating the downstream effector molecules (113, 114). Human pulmonary arterial hypertension (PAH) is associated with elevated Platelet-derived growth factor (PDGF)-BB and Transforming growth factor (TGF)-b1 levels (115). These factors also promote the proliferation of VSMCs and ECs. *In vivo* studies showed that intravenous DNAzymes suppressed pulmonary vasculature remodeling, such as the development of occlusive neointimal lesions, and decreased PAH progression by downregulating EGR1 and lowering PDGF-BB and TGF-b1 level. Thus, *Egr-1*-targeting DNAzymes are involved in the initiation and progression of pulmonary vascular remodeling in flow-related pulmonary hypertension and could serve as a potential target for PAH therapy in the future (116).

### 3.3. Oligonucleotide decoys target the EGR1 protein

**Figure 3:**
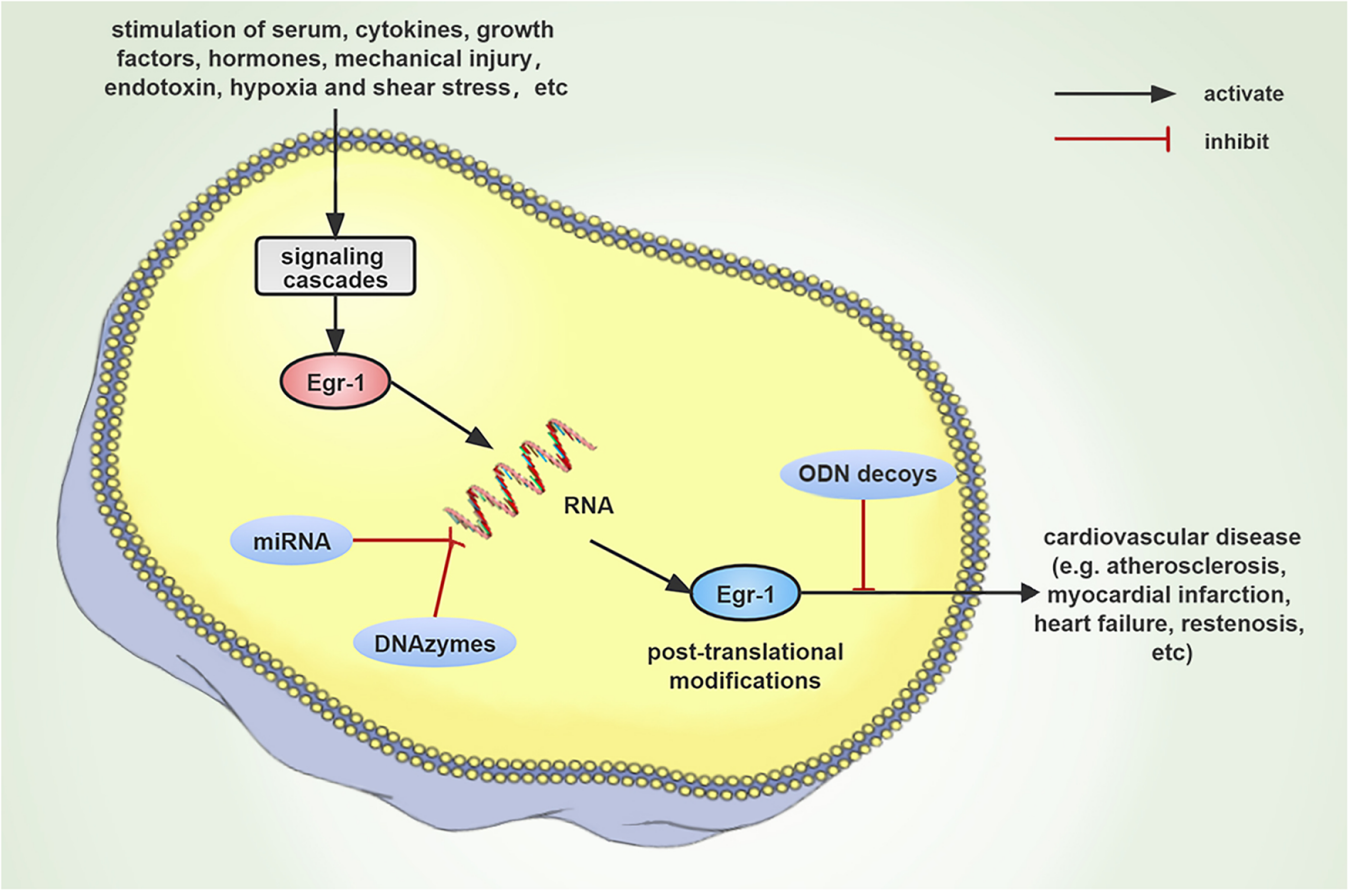
Schematic diagram that describes the progression of cardiovascular disease occurring due to the induction of *Egr-1* expression by numerous stimuli. Since *Egr-1* plays a major regulatory function in cardiovascular disease, it could be anticipated that *Egr-1* targeting DNAzymes, miRNAs, and ODNs decoy strategies could emerge as new therapeutic targets.

Synthetic double-stranded decoy oligonucleotides (ODNs) have also been used to target EGR1 at the protein level. In this technique, ODNs containing the DNA-binding component of the transcription factor bind to the DNA and inhibit the function of the transcription factor. ODNs have been utilized to prevent the expression of some genes. ODNs target particular regions of selected mRNAs and impede translation through base-pair hybridization, thus preventing the synthesis of a particular protein (117, 118). Studies have investigated the increasing effect of the sense and antisense oligodeoxynucleotides on the agonist-induced *Egr-1* mRNA and proteins. ODNs block the induction of VSMCs by PDGF-BB and angiotensin II. In one study, the carotid arteries in rabbits were damaged with a balloon, and the results showed that there was a decrease in vascular inflammation and neointimal hyperplasia. They also showed that elevated expression of the EGR1-dependent genes was suppressed by transfection of decoy ODNs targeting EGR1 (119). In another study, EGR1 decoy ODNs were designed and synthesized. They were then transfected into balloon-damaged carotid arteries and primary cultures of rat VSMCs, and their ability to bind to Egr-1 was assessed. The competitive binding of Egr-1 decoy ODNs to EGR1 lowered EGR1 level, which was mediated *via* cell proliferation-related genes such as *cyclin D1*, *CDK4*, and *PCNA*. This further inhibited neointimal hyperplasia and lowered the formation of VSMCs in rats with balloon-damaged arteries (120). Additional research has demonstrated that decoy strategies are quite efficient in the treatment of AMI and cardiac rejection (121, 122). Studies that were conducted using the pig and rat models indicated that NF-*κ*B decoy ODNs effectively suppressed neointimal development following arterial balloon injury. In an earlier study, the researchers could successfully transfect the NF-*κ*B decoy ODNs at the site of coronary stent placement. Their findings showed that these NF-*κ*B decoy ODNs exhibited a safe and beneficial effect on preventing restenosis after percutaneous coronary treatment since they prevented the apoptosis of ECs under hypoxia (121). To summarize, the use of EGR1 decoy ODNs as a novel therapy tool is critical for the prevention and treatment of coronary heart disease and restenosis after angioplasty (123, 124) (Figure 3).

## 4. Concluding remarks

In conclusion, studies conducted using *in vitro* tests, transgenic animal models, and human diseases revealed that cardiovascular diseases were caused by a variety of cellular and molecular pathways that affected the cell death processes in cardiac cells. *Egr-1* is an essential component of the cardiac cell death signaling pathways related to apoptosis, autophagy, mPOS, and ferroptosis. *Egr-1* is also involved in preserving heart homeostasis and assists in the development of cardiovascular disorders. The development of new signaling pathways and mediators in cardiovascular disease, as well as their role in tissue damage, has aided in the development of strategies for their specific targeting. Catalytic and non-catalytic nucleic acid approaches, such as DNAzymes, miRNAs, and ODNs decoys, have been used to investigate the primary regulatory role of Egr-1 in cardiovascular diseases, using animal models and *Egr-1*-deficient mice. In conclusion, *Egr-1* can be regarded as a potential therapeutic target for cardiovascular diseases.
